# E‐cadherin deregulation in breast cancer

**DOI:** 10.1111/jcmm.15140

**Published:** 2020-04-16

**Authors:** Giovanni Corso, Joana Figueiredo, Simone Pietro De Angelis, Federica Corso, Antonia Girardi, Joana Pereira, Raquel Seruca, Bernardo Bonanni, Patricia Carneiro, Gabriella Pravettoni, Elena Guerini Rocco, Paolo Veronesi, Giacomo Montagna, Virgilio Sacchini, Sara Gandini

**Affiliations:** ^1^ Division of Breast Surgery European Institute of Oncology IRCCS Milan Italy; ^2^ Department of Oncology and Hemato‐Oncology Faculty of Medicine University of Milan Milan Italy; ^3^ i3S ‐ Instituto de Investigação e Inovação em Saúde Universidade do Porto Porto Portugal; ^4^ IPATIMUP ‐ Institute of Molecular Pathology and Immunology University of Porto Porto Portugal; ^5^ Department of Experimental Oncology European Institute of Oncology IRCCS Milan Italy; ^6^ Faculty of Medicine University of Porto Porto Portugal; ^7^ Division of Cancer Prevention and Genetics European Institute of Oncology IRCCS Milan Italy; ^8^ Division of Applied Research Division for Cognitive and Psychological Science European Institute of Oncology IRCCS Milan Italy; ^9^ Division of Pathology European Institute of Oncology IRCCS Milan Italy; ^10^ Breast Service Department of Surgery Memorial Sloan Kettering Cancer Center New York NY USA

**Keywords:** breast cancer, breast cancer metastases, breast cancer prognosis, breast cancer survival, E‐cadherin

## Abstract

E‐cadherin protein (*CDH1* gene) integrity is fundamental to the process of epithelial polarization and differentiation. Deregulation of the E‐cadherin function plays a crucial role in breast cancer metastases, with worse prognosis and shorter overall survival. In this narrative review, we describe the inactivating mechanisms underlying *CDH1* gene activity and its possible translation to clinical practice as a prognostic biomarker and as a potential targeted therapy.

## 1 INTRODUCTION

The E‐cadherin (E‐cad) gene (*CDH1*) [OMIM + 192090] is a calcium‐dependent cell‐to‐cell adhesion molecule and tumour suppressor protein that is the only germline molecular defect associated with hereditary diffuse gastric and lobular breast cancers.^1^, ^2^ The E‐cad protein plays a critical role in establishing and maintaining polarized and differentiated epithelia through intercellular adhesion complexes. This molecule is considered an invasion suppressor, and its deregulation is often found in advanced cases of some epithelial carcinomas.^3^

Deletion or deregulation of E‐cad is also correlated with the infiltrative and metastatic ability of the tumour because of disruption of the cadherin‐catenin complex, with consequent loss of cell adhesion and concomitant increase in cell motility.^4^, ^5^ In gastric carcinoma in particular, defective mechanisms of E‐cad are well associated with cancer metastatization; patients carrying any somatic E‐cad alterations show the worst prognosis and shortest probability of overall survival (OS).^6^

In relation to breast cancer, regular E‐cad function presents as an inhibitor of metastasis. It has been shown that somatic E‐cad inactivation is associated with an aggressive pattern of breast cancer, particularly lymphovascular invasion and metastasis in the axillary lymph nodes.^7^, ^8^, ^9^, ^10^

Cancer metastatization often represents a dramatic event for the patient, due to the psychological impact of its worse prognosis and shorter OS. Prognostic molecular biomarkers are fundamental to assess the risk of tumour relapse after curative intent and cancer metastatization in this setting.

In this narrative review, we describe the principal mechanisms of E‐cad inactivation (genetic and epigenetic) and its possible clinical implications for breast cancer as a prognostic and therapeutic factor.

## 2 MECHANISMS OF E‐CAD INACTIVATION

Breast cancer's progression and capacity to invade and metastasize to distant sites is strongly associated with the loss of E‐cad. It has been postulated that loss of E‐cad expression is an early gatekeeper event in in situ lobular breast cancer and a precursor of invasive lobular breast cancer.^7^, ^11^ Over the last several years, a number of mechanisms have been identified as the cause of E‐cad inactivation in breast cancer.^12^, ^13^, ^14^ Inactivating mutations in the *CDH1* gene have been frequently described and can explain a great proportion of invasive lobular breast cancer cases. Indeed, one of the first studies addressing the contribution of *CDH1* mutations to the absence of E‐cad expression found that 55% (21 of 38 cases) of breast cancers from the lobular subtype harboured *CDH1* genetic alterations.^15^ In a subsequent study, Berx and colleagues^13^ found somatic mutations of the E‐cad gene at a comparable frequency: 56% (23 of 41) of lobular carcinoma cases analysed showed E‐cad mutations. This high frequency of *CDH1* mutations in breast cancer was not reported in all studies, however. Huiping et al^16^ examined 40 lobular breast cancers for E‐cad mutations and identified only 5 frameshift and 1 splice site mutations (constituting 15% of the cases in the study). In a different study by Droufakou et al,^12^ 6 novel mutations were detected in a set of 22 invasive lobular carcinomas, accounting for 27% of the cases analysed. Interestingly, in the majority of these studies, mutations were found in combination with loss of heterozygosity (LOH) at the E‐cad chromosomal locus, spotlighting E‐cad as a tumour suppressor gene.^12^, ^13^, ^16^ Specifically, among the 23 mutations identified by Berx and colleagues,^13^ 21 were found in combination with LOH of the wild type*CDH1l*ocus. These data were further validated by Huiping and colleagues,^16^ who detected LOH at 16q22.1 in all of the lobular breast tumours examined by their study. Currently, it is well established that 50% of invasive lobular carcinomas show LOH, which is determinant for protein dysfunction and loss of expression.^12^, ^13^, ^17^

Apart from structural genetic alterations, epigenetic modifications have also emerged as a possible cause for the impairment of E‐cad expression and function. In particular, hypermethylation of the *CDH1* promoter is the predominant mechanism of E‐cad loss in multiple types of cancer, including breast cancer.^18^, ^19^, ^20^ In this context, Shargh and colleagues^21^ investigated the association between *CDH1* promoter methylation and E‐cad expression in 50 ductal breast cancer cases and their respective paired normal breast samples. They observed that 94% of ductal breast cancers were methylated at the *CDH1* promoter. Furthermore, they found that there was no detectable E‐cad expression in all the cases displaying complete promoter methylation.

Clinically, an association between *CDH1* methylation and breast cancer progression has also been reported. Nass et al^22^ examined 111 cases of ductal breast carcinomas and observed that *CDH1* methylation was present in 31% of in situ lesions, in 52% of invasive tumours, and in 61% of metastatic cancers, indicating an increase of CpG island methylation as cells gain invasiveness and metastatic potential. Later on, the impact of hypermethylation on 6 tumour suppressor genes in tumour progression was evaluated using a series of 151 primary breast tumours, in which the *CDH1* promoter was found to be hypermethylated in 53% of cases. Strikingly, in cases with sentinel lymph node metastasis, *CDH1* was the most frequently methylated gene (90%), reinforcing the evidence that *CDH1* hypermethylation prevails at a more advanced disease stage.^23^ Sebova and colleagues^24^ proposed that *CDH1* hypermethylation can be used as a biomarker for potentially metastasizing tumours. They observed that *CDH1* promoter hypermethylation was preferentially observed in breast cancer cases with positive lymph node metastasis and in cases from more aggressive immunohistochemical subtypes. A correlation between *CDH1* methylation status and the prognosis of breast cancer patients was also explored using a series of 137 primary breast cancers, 85 matched normal breast tissue samples, and 13 lung metastasis cases. It was observed that 40.9% of breast cancers and 61.5% of lung metastasis samples were hypermethylated in the *CDH1* promoter, while none of the normal breast samples displayed *CDH1* methylation. In addition, patients with *CDH1* methylation presented significantly poor OS as well as lower disease‐free survival (DFS), supporting the significance of *CDH1* expression as a predictive biomarker of poor prognosis in breast cancer.^25^

At the transcriptional level, several molecules are known to bind to specific DNA sequences of the E‐boxes of the *CDH1* promoter, repressing the transcription of E‐cad and activating mesenchymal genes, and thus promoting the so called ‘epithelial to mesenchymal transition’ (EMT).^26^ This loss of epithelial gene expression and activation of a mesenchymal molecular profile can involve SNAIL, zinc finger E‐box‐binding (ZEB) and TWIST transcription factors, whose expression is critical to cancer development.^27^, ^28^ In breast cancer, it has been reported that silencing of SNAIL increases E‐cad expression and, consequently, decreases expression of mesenchymal markers, decreases tumourigenicity and inhibits the invasive behaviour of breast cancer cells.^29^ Moreover, Xiang and colleagues^30^ studied the expression of ZEB1 by immunohistochemistry in 102 breast carcinoma samples and found that carcinomas with high aggressive potential presented high levels of ZEB1. They were able to associate increased levels of ZEB1 with lymph node metastasis and advanced disease stage, proposing ZEB1 as an additional prognostic marker in breast carcinoma. A microarray gene expression data set from 57 invasive human breast tumours also revealed that 70% of invasive lobular carcinomas, 32% of invasive ductal carcinomas, and 30% of mixed ductal/lobular carcinomas presented higher expression of TWIST when compared with normal breast tissue, awarding TWIST an important role in tumour metastasis.^31^

**Figure 1:**
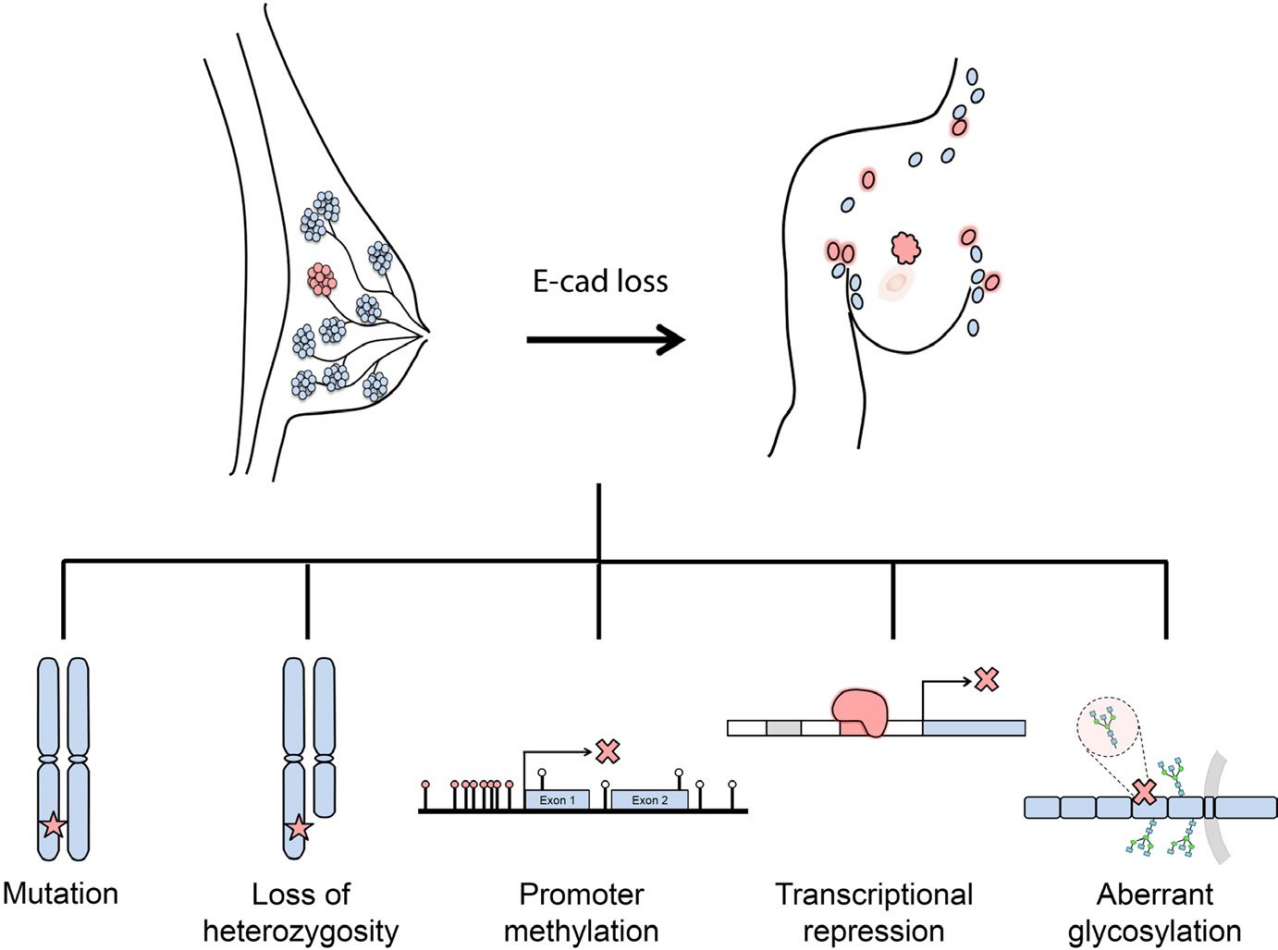
Mechanisms underlying E‐cad inactivation in breast cancer. Loss of E‐cad expression and the spreading abilities of breast cancer cells have been associated with mutations in *CDH1* gene, loss of heterozygosity at the E‐cad chromosomal locus, hypermethylation of the *CDH1* promoter, transcriptional repression and post‐translational modifications, such as aberrant glycosylation

More recently, post‐translational mechanisms, such as glycosylation, have emerged as critical processes in cancer. Aberrant glycosylation has itself been suggested as a new hallmark of cancer, since it impacts cell differentiation, adhesion and proliferation.^31^ In particular, E‐cad can be post‐translationally modified by O (oxygen)‐ and N (nitrogen)‐glycosylation.^32^, ^33^ These modifications have been reported to be essential for E‐cad folding, trafficking and stability at the membrane.^34^, ^35^, ^36^, ^37^ Aberrant O‐glycosylation of E‐cad in its cytoplasmic domain blocks its exocytosis to the cell surface, inhibiting E‐cad‐mediated cell adhesion.^38^ Furthermore, O‐glycosylation increases cell migration and metastasis mostly by decreasing E‐cad at the surface of breast cancer cells.^39^ In contrast to O‐glycosylation, which occurs at the intracellular portion of E‐cad, N‐glycosylation takes place in 4 possible sites at the extracellular domain responsible for homophilic interaction between 2 cadherin molecules of adjacent cells.^33^ Thus, Zhao and colleagues^40^ undertook a detailed study of the role of N‐glycosylation in E‐cad adhesive function and stability at the membrane. They demonstrated that the removal of glycans in specific positions reduces protein stability and modifies the composition of adherens junctions, impairing cell compaction. Supporting the relevance of glycolsylation in breast cancer, it was demonstrated that GCNT2—a gene‐encoding glucosaminyl (N‐acetyl) transferase 2 that plays a critical role in glycosylation—is related to basal‐like and metastatic phenotypes in both breast cancer cell lines of human and mouse origins, and of human breast tumour samples. GCNT2 is overexpressed in highly metastatic breast cancer, and its expression correlates with adverse pathological features and progression of disease. Remarkably, it has been suggested that the GCNT2's metastasis‐promoting effect is mediated through modulation of E‐cad protein levels: the overexpression of GCNT2 decreases E‐cad expression, whereas its down‐regulation induces increased levels of the protein.^41^ Despite the low number of studies addressing the impact of the E‐cad glycosylation pattern in breast cancer, it is clear that post‐translational modifications can underlie the spreading abilities of breast cancer cells associated with E‐cad dysfunction. As such, abnormal glycan structures are becoming attractive in clinics as potential therapeutic targets or prognostic biomarkers. Figure 1 provides an illustration of the mechanisms underlying E‐cad inactivation in breast cancer.

## 3 E‐CAD AS PROGNOSTIC BIOMARKER

Breast cancer is a highly heterogeneous disease in which even similar clinical and pathologic features lead to distinct outcomes. These observations indicate that staging systems based on clinical and pathologic findings may have reached their limit of usefulness and impelled the need for molecular biomarkers—as an added value—to predict patients' outcome and treatment. Tumour size, lymph node status, histological grade, and oestrogen receptor (ER), progesterone receptor (PR) and HER2 expression are currently evaluated as parameters to define therapy and prognosis in breast cancer patients. Although these factors are still essential, they do not predict breast cancer prognosis with high accuracy. Novel and molecular biomarkers are required to improve survival rates for these patients.

E‐cad is a fundamental intercellular adhesion molecule that links with catenins forming the E‐cad/β‐catenin/α‐catenin complex. This molecule interacts with the actin cytoskeleton,^4^ which stabilizes cell interaction, cell polarity and the integrity of epithelial tissue. Disruption of E‐cad causes alteration of the intercellular junction, and subsequently increases cell migration ability, tumour invasion and metastasis. In detail, in a homeostatic situation, E‐cadherin is expressed at the adherens junctions playing a crucial role in cell‐cell adhesion and in the polarized architecture of the tissue. *CDH1* mutations can, however, induce loss of E‐cadherin function and abnormally activate a number of mechanisms and signalling pathways. Mutated proteins present severe structural abnormalities, resulting in protein misfolding that is recognized and degraded by endoplasmic reticulum‐associated degradation (ERAD).^42^ At the plasma membrane, mutant proteins cannot establish the cytoplasmic catenin complex, allowing its rapid internalization and degradation.^43^ E‐cadherin loss results in abnormal activation of EGFR and Notch pathways, with consequences on cell motility, invasion and resistance to apoptotic stimuli.^3^

Several studies have evaluated the potential clinical implications of E‐cad inactivation in breast cancer. A recent meta‐analysis comprising 33 retrospective studies including 7353 breast cancer patients evaluated the association of E‐cad and OS, DFS and clinicopathologic factors in breast cancer.^44^ In this study, reduced E‐cad expression on membrane was significantly associated with OS (hazard ratio [HR] 1.57, 95% confidence interval [CI] 1.17‐2.10) and DFS (HR 1.37, 95% CI 1.07‐1.75). Furthermore, the down‐regulated expression of E‐cad was correlated with tumour size, lymph node status, TNM stage and histological grade. In detail, E‐cad low expression was significantly associated with lymph node status (positive vs negative: odds ratio [OR] 1.55, 95% CI 1.15‐2.10), tumour size (≥2 cm vs <2 cm, OR 1.38, 95% CI 1.18‐1.60), histological grade (II–III vs I: OR 1.44, 95% CI 1.06‐1.96) and TNM stage (T3/T4 vs T1/T2: OR 2.44, 95% CI 1.75‐3.41).

These results herein reported may change the paradigm thus far described for somatic E‐cad deficiency. The most striking finding was that patients with E‐cad low expression (presenting tumours with structural alterations) had the worst OS. Molecular variables such as *CDH1* alterations may be crucial to predict the survival of breast cancer patients.^8^

The presence of *CDH1* epigenetic and structural alterations in a diagnostic/pre‐operative biopsy may provide clinically useful information to improve patient management, particularly to infer the prognosis of breast cancer and the pattern of tumour dissemination.

## 4 POTENTIAL ROLE OF E‐CAD AS A THERAPEUTIC TARGET

To date, E‐cad is not a molecular target for therapeutic intervention; however, in vitro studies have demonstrated that germline E‐cad function can be restored upon targeted treatment. Important novel E‐cad cross‐talk mechanisms are described. Growth factor signals are hyperactivated upon E‐cad loss, regardless of somatic activating mutations in downstream effectors. In particular, the PI3K/Akt pathway is activated upon E‐cad loss in the absence of specific oncogenic mutations. Interestingly, it has been demonstrated in vitro that lobular breast cancer cells are sensitive to pharmacological inhibition of Akt, using ATP competitor AZD5363 and two allosteric inhibitors, MK2206 and VIII. The strongest reduction in term of growth and survival for cancer cells was observed for MK2206 Akt inhibitor.^45^ Recently, Bajrami et al^46^ identified a synthetic lethal interaction between E‐cad and *ROS1*. *ROS1* gene rearrangements represent well‐known cancer‐related aberrations and therapeutic targets of approved drugs. The Authors showed that *ROS1* inhibition in E‐cad‐defective breast tumour cell lines and patient‐derived breast tumour xenografts resulted in tumour cell death. This evidence suggests a possible role for *ROS1* inhibitors in the treatment of patients with E‐cad‐defective tumours and may represent a foundational element for future ad hoc translational studies and clinical trials. Accordingly, a phase II study evaluating the effect of the combination of *ROS1*‐targeting drugs crizotinib and fulvestrant in advanced E‐cad negative, lobular breast cancer or diffuse gastric cancer is ongoing.^47^

The heterodimer E‐cad‐epidermal growth factor receptor (EGFR) complex is another attractive therapeutic target. The presence of extracellular E‐cad mutations disturbs the stability of E‐cad‐EGFR heterodimers, allowing for receptor activation by the ligand and consequent activation of the RhoA signalling pathway, accompanied by enhanced cell motility. Upon interaction with EGFR, E‐cad exerts an inhibitory function that modulates the kinase activity of the receptor in an adhesion‐independent manner. The extracellular E‐cad mutants, by reducing its affinity for EGFR, increase the fraction of unbound EGFR, which can thus be activated, resulting in enhanced cell motility. This effect is transmitted through the activation of RhoA.^48^ Further study is needed to clarify the role of HER2 in this pathway and the effect of targeted treatment using HER2 inhibitors (ie trastuzumab).

## 5 HOW TO MANAGE E‐CAD IN THE CLINICAL SETTING

E‐cad may play a potential strategic role in the clinical management of breast cancer patients as a predictor of prognosis and survival. In this review, we analyzed several studies reporting that E‐cad dysfunction is associated with worse prognosis and shorter OS. Although mechanisms of *CDH1* inactivation are still complex to manage routinely, it is reasonable to evaluate E‐cad expression in breast tumours using standard methods, such as immunohistochemistry. We have already demonstrated that CDH1 inactivation is a suitable predictive biomarkers of prognosis in gastric cancer's patients. In particular, the worst patient survival rate among all cases analysed was seen in patients with tumours carrying CDH1 structural alterations.^6^

**Figure 2:**
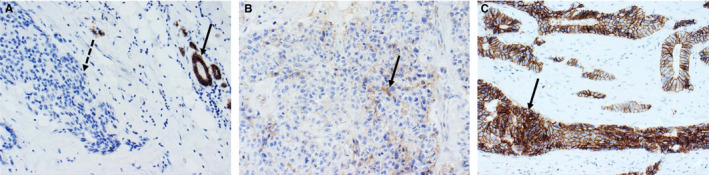
Differences in E‐cad immunoreactivity in breast cancer. Representative micrographs of lobular carcinoma with no immunohistochemical expression of E‐cad (dashed arrow in A, left) and adjacent normal ducts with normal strong membranous E‐cad staining (full arrow in A, right); invasive breast cancers, no special type showing partial loss (B) and strong (C) membranous immunoreactivity for E‐cad. Original magnification 200×. E‐cad, E‐cadherin

**Figure 3:**
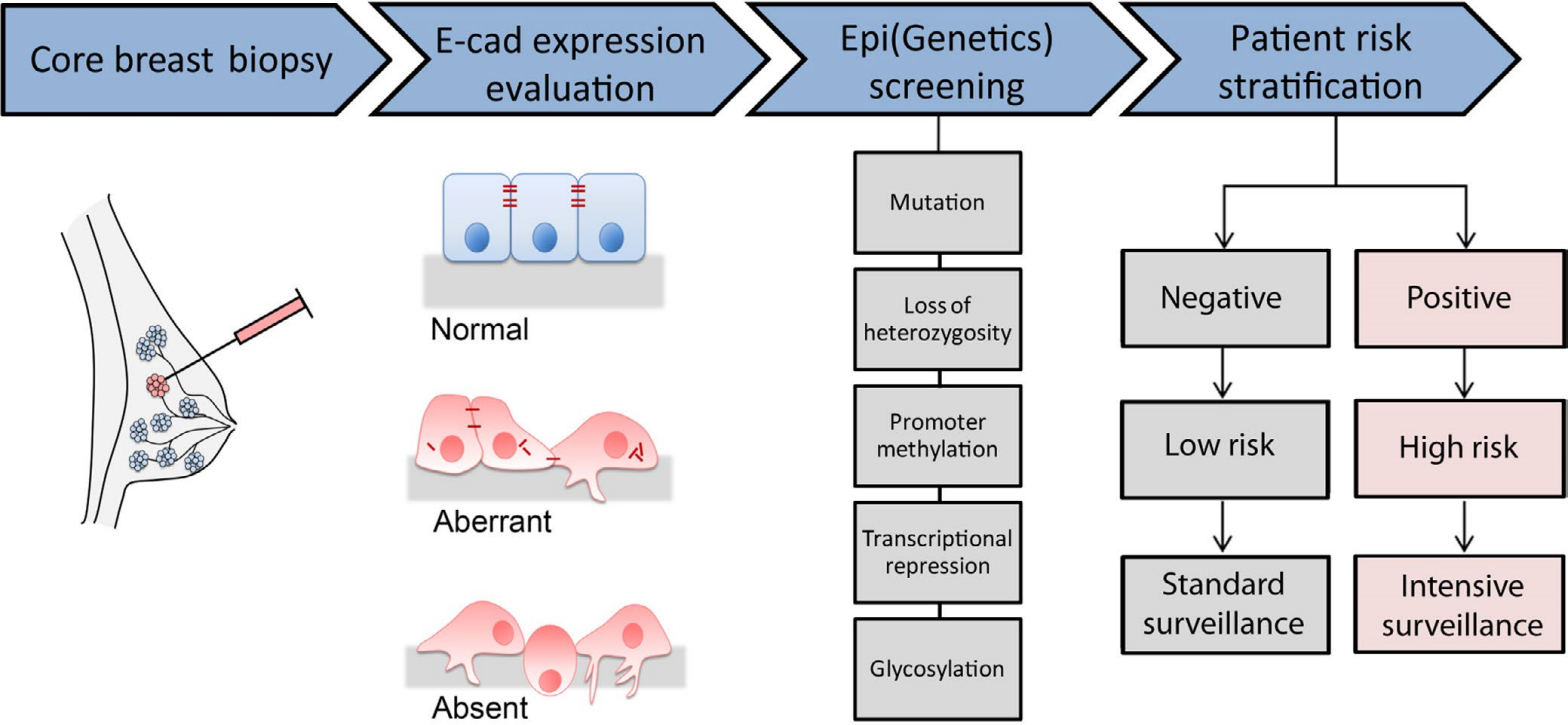
Core biopsy is the principal approach for breast cancer diagnosis. Histopathology and immunohistochemistry are performed on breast biopsy; E‐cad expression is classified as ‘normal’, ‘aberrant’ or ‘absent’. Genetic or epigenetic mechanisms are underlying aberrant or absent E‐cad expression. Patients carrying any somatic genetic/epigenetic defect are classified as ‘high risk’ for breast cancer and carry a worse prognosis. A personalized and intensive screening (follow‐up) is proposed for those patients for early identification of any breast cancer relapse

In normal breast tissue, epithelial cells show strong and complete membranous expression of E‐cad.^49^ Among breast cancers, nearly 90% of invasive lobular carcinomas display complete or partial loss of E‐cad immunohistochemical expression that is considered an important (but not necessary) diagnostic feature for this histological subtype (Figure 2).^50^, ^51^, ^52^ However, aberrant E‐cad immunoreactivity such as complete absence or reduced membranous staining, and punctate, or cytoplasmic expression have been observed in other breast cancer subtypes.^53^ Given the absence of standardized methods, different studies have used variable antibodies and scoring criteria for the immunohistochemical analysis of E‐cad. These variabilities may subtend the differences observed in the clinical relevance of aberrant E‐cad expression.^44^, ^54^ Indeed, the validation of a robust and reproducible method for the assessment of immunohistochemical expression of E‐cad is fundamental to translate these findings to the clinical setting. In this scenario, aberrant/negative immunohistochemical expression may lead to the evaluation of genetic and epigenetic factors. Breast cancer patients carrying any somatic alterations would then be classified as ‘high risk’ for breast cancer relapse. A multidisciplinary team can organize a personalized follow‐up, aiming to reduce breast cancer mortality and improve prognosis (Figure 3).

## 6 CONCLUSION

In this narrative article, we have provided evidence for the aggressive patterning of E‐cad deficiency in breast cancer metastatization. This pathway may influence the staging and treatment of patients with breast cancer.

There currently is a substantial level of interest in the combination of molecular targeting and prognostic biomarkers for patients with clinically worse disease. Integrated clinical and molecular staging will ultimately serve, in the near future, as the basis for generation of informed treatment decisions along the entire prognosis spectrum. Eventually, this and other related studies will help clinicians to identify patients with tumours who have potential worse prognoses and to identify the clinical scenarios in which targeted therapies should be combined with ‘traditional’ agents with the goal of curing greater numbers of patients with breast cancer.

## CONFLICT OF INTEREST

The authors have no potential conflicts of interest to declare.

## AUTHOR CONTRIBUTIONS

GC, SPD, FC, AG, AI, EGR and SG researched data for this article. GC, JF, SPD, FC, AG, AI, JP, RS, BB, PC, GP, EGR, PV, GM, VS and SG made substantial contributions to the discussion of content. GC, JF and EGR wrote the manuscript. All authors reviewed and/or edited the manuscript before submission.

## Data Availability

The data that support the findings of this study are available from the corresponding author upon reasonable request.

## References

[1] Caldas C , Carneiro F , Lynch HT , et al. Familial gastric cancer: overview and guidelines for management. J Med Genet. 1999;36:873‐880. 10593993 PMC1734270

[2] Corso G , Figueiredo J , La Vecchia C , et al. Hereditary lobular breast cancer with an emphasis on E‐cadherin genetic defect. J Med Genet. 2018;55:431‐441. 29929997 10.1136/jmedgenet-2018-105337

[3] Takeichi M . Cadherins in cancer: implications for invasion and metastasis. Curr Opin Cell Biol. 1993;5:806‐811. 8240824 10.1016/0955-0674(93)90029-p

[4] Christofori G , Semb H . The role of the cell‐adhesion molecule E‐cadherin as a tumour‐suppressor gene. Trends Biochem Sci. 1999;24:73‐76. 10098402 10.1016/s0968-0004(98)01343-7

[5] Van Aken E , De Wever O , Correia da Rocha AS , Mareel M . Defective E‐cadherin/catenin complexes in human cancer. Virchows Arch. 2001;439:725‐751. 11787845 10.1007/s004280100516

[6] Corso G , Carvalho J , Marrelli D , et al. Somatic mutations and deletions of the E‐cadherin gene predict poor survival of patients with gastric cancer. J Clin Oncol. 2013;31:868‐875. 23341533 10.1200/JCO.2012.44.4612

[7] Hunt NC , Douglas‐Jones AG , Jasani B , Morgan JM , Pignatelli M . Loss of E‐cadherin expression associated with lymph node metastases in small breast carcinomas. Virchows Arch. 1997;430:285‐289. 9134039 10.1007/BF01092751

[8] Li P , Sun T , Yuan Q , Pan G , Zhang J , Sun D . The expressions of NEDD9 and E‐cadherin correlate with metastasis and poor prognosis in triple‐negative breast cancer patients. Onco Targets Ther. 2016;9:5751‐5759. 27703373 10.2147/OTT.S113768 PMC5036611

[9] Liu JB , Feng CY , Deng M , et al. E‐cadherin expression phenotypes associated with molecular subtypes in invasive non‐lobular breast cancer: evidence from a retrospective study and meta‐analysis. World J Surg Oncol. 2017;15:139. 28764784 10.1186/s12957-017-1210-8 PMC5539617

[10] Yang L , Wang XW , Zhu LP , et al. Significance and prognosis of epithelial‐cadherin expression in invasive breast carcinoma. Oncol Lett. 2018;16:1659‐1665. 30008850 10.3892/ol.2018.8836 PMC6036376

[11] Oka H , Shiozaki H , Kobayashi K , et al. Expression of E‐cadherin cell adhesion molecules in human breast cancer tissues and its relationship to metastasis. Cancer Res. 1993;53:1696‐1701. 8453644

[12] Droufakou S , Deshmane V , Roylance R , Hanby A , Tomlinson I , Hart IR . Multiple ways of silencing E‐cadherin gene expression in lobular carcinoma of the breast. Int J Cancer. 2001;92:404‐408. 11291078 10.1002/ijc.1208

[13] Berx G , Cleton‐Jansen AM , Nollet F , et al. E‐cadherin is a tumour/invasion suppressor gene mutated in human lobular breast cancers. EMBO J. 1995;14:6107‐6115. 8557030 10.1002/j.1460-2075.1995.tb00301.x PMC394735

[14] Hanby AM , Hughes TA . In situ and invasive lobular neoplasia of the breast. Histopathology. 2008;52:58‐66. 18171417 10.1111/j.1365-2559.2007.02891.x

[15] De Leeuw WJ , Berx G , Vos CB , et al. Simultaneous loss of E‐cadherin and catenins in invasive lobular breast cancer and lobular carcinoma in situ. J Pathol. 1997;183:404‐411. 9496256 10.1002/(SICI)1096-9896(199712)183:4<404::AID-PATH1148>3.0.CO;2-9

[16] Huiping C , Sigurgeirsdottir JR , Jonasson JG , et al. Chromosome alterations and E‐cadherin gene mutations in human lobular breast cancer. Br J Cancer. 1999;81(7):1103‐1110. 10584868 10.1038/sj.bjc.6690815 PMC2374316

[17] Berx G , Cleton‐Jansen AM , Strumane K , et al. E‐cadherin is inactivated in a majority of invasive human lobular breast cancers by truncation mutations throughout its extracellular domain. Oncogene. 1996;13(18):1919‐1925. 8934538

[18] Mielnicki LM , Asch HL , Asch BB . Genes, chromatin, and breast cancer: an epigenetic tale. J Mammary Gland Biol Neoplasia. 2001;6:169‐182. 11501577 10.1023/a:1011356623442

[19] Machado JC , Oliveira C , Carvalho R , et al. E‐cadherin gene (CDH1) promoter methylation as the second hit in sporadic diffuse gastric carcinoma. Oncogene. 2001;20:1525‐1528. 11313896 10.1038/sj.onc.1204234

[20] Bhagat R , Premalata CS , Shilpa V , et al. Altered expression of beta‐catenin, E‐cadherin, and E‐cadherin promoter methylation in epithelial ovarian carcinoma. Tumour Biol. 2013;34:2459‐2468. 23605324 10.1007/s13277-013-0797-9

[21] Shargh SA , Sakizli M , Khalaj V , et al. Downregulation of E‐cadherin expression in breast cancer by promoter hypermethylation and its relation with progression and prognosis of tumor. Med Oncol. 2014;31:250. 25260805 10.1007/s12032-014-0250-y

[22] Nass SJ , Herman JG , Gabrielson E , et al. Aberrant methylation of the estrogen receptor and e‐cadherin 5′ CpG islands increases with malignant progression in human breast cancer. Cancer Res. 2000;60:4346. 10969774

[23] Shinozaki M , Hoon DS , Giuliano AE , et al. Distinct hypermethylation profile of primary breast cancer is associated with sentinel lymph node metastasis. Clin Cancer Res. 2005;11:2156. 15788661 10.1158/1078-0432.CCR-04-1810

[24] Sebova K , Zmetakova I , Bella V , et al. RASSF1A and CDH1 hypermethylation as potential epimarkers in breast cancer. Cancer Biomark. 2011;10:13‐26. 22297548 10.3233/CBM-2012-0230 PMC13016238

[25] Liu J , Sun X , Qin S , et al. CDH1 promoter methylation correlates with decreased gene expression and poor prognosis in patients with breast cancer. Oncol Lett. 2016;11:2635‐2643. 27073531 10.3892/ol.2016.4274 PMC4812319

[26] Peinado H , Olmeda D , Cano A . Snail, Zeb and bHLH factors in tumour progression: an alliance against the epithelial phenotype? Nat Rev Cancer. 2007;7:415. 17508028 10.1038/nrc2131

[27] Lamouille S , Xu J , Derynck R . Molecular mechanisms of epithelial–mesenchymal transition. Nat Rev Mol Cell Biol. 2014;15:178. 24556840 10.1038/nrm3758 PMC4240281

[28] Paredes J , Figueiredo J , Albergaria A , et al. Epithelial E‐ and P‐cadherins: role and clinical significance in cancer. Biochim Biophys Acta. 2012;1826:297‐311. 22613680 10.1016/j.bbcan.2012.05.002

[29] Olmeda D , Moreno‐Bueno G , Flores JM , Fabra A , Portillo F , Cano A . SNAI1 is required for tumor growth and lymph node metastasis of human breast carcinoma MDA‐MB‐231 cells. Cancer Res. 2007;67:1721. 10.1158/0008-5472.CAN-07-2318 18089802

[30] Xiang S , Liu YM , Chen X , et al. ZEB1 expression is correlated with tumor metastasis and reduced prognosis of breast carcinoma in Asian patients. Cancer Invest. 2015;33:225‐231. 25950745 10.3109/07357907.2015.1022258

[31] Vajaria BN , Patel PS . Glycosylation: a hallmark of cancer? Glycoconjugate J. 2017;34:147‐156. 10.1007/s10719-016-9755-2 27975160

[32] Pinho SS , Reis CA , Paredes J , et al. The role of N‐acetylglucosaminyltransferase III and V in the post‐transcriptional modifications of E‐cadherin. Hum Mol Genet. 2009;18:2599‐2608. 19403558 10.1093/hmg/ddp194

[33] Pinho SS , Seruca R , Gärtner F , et al. Modulation of E‐cadherin function and dysfunction by N‐glycosylation. Cell Mol Life Sci. 2011;68:1011‐1020. 21104290 10.1007/s00018-010-0595-0 PMC11114786

[34] Pinho SS , Carvalho S , Marcos‐Pinto R , et al. Gastric cancer: adding glycosylation to the equation. Trends Mol Med. 2013;19:664‐676. 23932995 10.1016/j.molmed.2013.07.003

[35] Pinho SS , Figueiredo J , Cabral J , et al. E‐cadherin and adherens‐junctions stability in gastric carcinoma: functional implications of glycosyltransferases involving N‐glycan branching biosynthesis, N‐acetylglucosaminyltransferases III and V. Biochim Biophys Acta. 2013;1830:2690‐2700. 23671930 10.1016/j.bbagen.2012.10.021

[36] Zhou F , Su J , Fu L , et al. Unglycosylation at Asn‐633 made extracellular domain of E‐cadherin folded incorrectly and arrested in endoplasmic reticulum, then sequentially degraded by ERAD. Glycoconj J. 2008;25:727‐740. 18491227 10.1007/s10719-008-9133-9

[37] Liwosz A , Lei T , Kukuruzinska MA . N‐glycosylation affects the molecular organization and stability of E‐cadherin junctions. J Biol Chem. 2006;281:23138‐23149. 16682414 10.1074/jbc.M512621200

[38] Zhu W , Leber B , Andrews DW . Cytoplasmic O‐glycosylation prevents cell surface transport of E‐cadherin during apoptosis. EMBO J. 2001;20:5999. 11689440 10.1093/emboj/20.21.5999 PMC125709

[39] Gu Y , Mi W , Ge Y , et al. GlcNAcylation plays an essential role in breast cancer metastasis. Cancer Res. 2010;70:6344. 20610629 10.1158/0008-5472.CAN-09-1887

[40] Zhao H , Liang Y , Xu Z , et al. N‐Glycosylation affects the adhesive function of E‐Cadherin through modifying the composition of adherens junctions (AJs) in human breast carcinoma cell line MDA‐MB‐435. J Cell Biochemi. 2008;104:162‐175. 10.1002/jcb.21608 17979184

[41] Zhang H , Meng F , Wu S , et al. Engagement of I‐branching {beta}‐1, 6‐N‐acetylglucosaminyltransferase 2 in breast cancer metastasis and TGF‐{beta} signaling. Cancer Res. 2011;71:4846‐4856. 21750175 10.1158/0008-5472.CAN-11-0414 PMC3903410

[42] Simões‐Correia J , Figueiredo J , Oliveira C , van Hengel J , Seruca R , van Roy F , Suriano G . Endoplasmic reticulum quality control: a new mechanism of E‐cadherin regulation and its implication in cancer. Hum Mol Genet. 2008;17:3566‐3576. 18772194 10.1093/hmg/ddn249

[43] Figueiredo J , Söderberg O , Simões‐Correia J , Grannas K , Suriano G , Seruca R . The importance of E‐cadherin binding partners to evaluate the pathogenicity of E‐cadherin missense mutations associated to HDGC. Eur J Hum Genet. 2013;21:301‐309. 22850631 10.1038/ejhg.2012.159 PMC3573198

[44] Li Z , Yin S , Zhang L , Liu W , Chen B . Prognostic value of reduced E‐cadherin expression in breast cancer: a meta‐analysis. Oncotarget. 2017;8:16445‐16455. 28147315 10.18632/oncotarget.14860 PMC5369975

[45] Teo K , Gómez‐Cuadrado L , Tenhagen M , et al. E‐cadherin loss induces targetable autocrine activation of growth factor signalling in lobular breast cancer. Sci Rep. 2018;8:15454. 30337563 10.1038/s41598-018-33525-5 PMC6193986

[46] Bajrami I , Marlow R , van de Ven M , et al. E‐cadherin/ROS1 inhibitor synthetic lethality in breast cancer. Cancer Discov. 2018;8:498‐515. 29610289 10.1158/2159-8290.CD-17-0603 PMC6296442

[47] US National Library of Medicine . ROS1 Targeting with Crizotinib in Advanced E‐cadherin Negative, ER Positive Lobular Breast Cancer or Diffuse Gastric Cancer Study (ROLo). ClinicalTrials.gov Identifier: NCT03620643; 2019.

[48] Mateus AR , Simões‐Correia J , Figueiredo J , et al. E‐cadherin mutations and cell motility: a genotype‐phenotype correlation. Exp Cell Res. 2009;315:1393‐1402. 19268661 10.1016/j.yexcr.2009.02.020

[49] Kowalski PJ , Rubin MA , Kleer CG . E‐cadherin expression in primary carcinomas of the breast and its distant metastases. Breast Cancer Res. 2003;5:R217‐R222. 14580257 10.1186/bcr651 PMC314411

[50] Dabbs DJ , Schnitt SJ , Geyer FC , et al. Lobular neoplasia of the breast revisited with emphasis on the role of E‐cadherin immunohistochemistry. Am J Surg Pathol. 2013;37:e1‐11. 23759937 10.1097/PAS.0b013e3182918a2b

[51] McCart Reed AE , Kutasovic JR , Lakhani SR , Simpson PT . Invasive lobular carcinoma of the breast: morphology, biomarkers and 'omics. Breast Cancer Res. 2015;17:12. 25849106 10.1186/s13058-015-0519-x PMC4310190

[52] Morrogh M , Andrade VP , Giri D , et al. Cadherin‐catenin complex dissociation in lobular neoplasia of the breast. Breast Cancer Res Treat. 2012;132:641‐652. 22080244 10.1007/s10549-011-1860-0 PMC4349355

[53] Jansen M, Wright NA, eds. Stem Cells, Pre‐neoplasia, and Early Cancer of the Upper Gastrointestinal Tract (Advances in Experimental Medicine and Biology Book 908). Switzerland: Springer; 2016.

[54] Horne HN , Oh H , Sherman ME , et al. E‐cadherin breast tumor expression, risk factors and survival: pooled analysis of 5,933 cases from 12 studies in the Breast Cancer Association Consortium. Sci Rep. 2018;8:6574. 29700408 10.1038/s41598-018-23733-4 PMC5920115

